# Low MxA Expression Predicts Better Immunotherapeutic Outcomes in Glioblastoma Patients Receiving Heat Shock Protein Peptide Complex 96 Vaccination

**DOI:** 10.3389/fonc.2022.865779

**Published:** 2022-07-12

**Authors:** Yi Wang, Chunzhao Li, Xiaohan Chi, Xijian Huang, Hua Gao, Nan Ji, Yang Zhang

**Affiliations:** ^1^ Department of Neurosurgery, Beijing Tiantan Hospital, Capital Medical University, Beijing, China; ^2^ China National Clinical Research Center for Neurological Diseases, Beijing Tiantan Hospital, Capital Medical University, Beijing, China; ^3^ Beijing Advanced Innovation Center for Big Data-Based Precision Medicine, Beihang University, Beijing, China; ^4^ Cure & Sure Biotech Co., LTD, Shenzhen, China

**Keywords:** glioblastoma, MxA, gp96, cancer treatment vaccine, biomarker, immune cell infiltration

## Abstract

Heat shock protein peptide complex 96 (HSPPC-96) has been proven to be a safe and preliminarily effective therapeutic vaccine in treating newly diagnosed glioblastoma multiforme (GBM) (NCT02122822). However, the clinical outcomes were highly variable, rendering the discovery of outcome-predictive biomarkers essential for this immunotherapy. We utilized multidimensional immunofluorescence staining to detect CD4^+^ CD8^+^ and PD-1^+^ immune cell infiltration levels, MxA and gp96 protein expression in pre-vaccination GBM tissues of 19 patients receiving HSPPC-96 vaccination. We observed low MxA expression was associated with longer OS than high MxA expression (48 months vs. 20 months, p=0.038). Long-term survivors (LTS) exhibited significantly lower MxA expression than short-term survivors (STS) (p= 0.0328), and ROC curve analysis indicated MxA expression as a good indicator in distinguishing LTS and STS (AUC=0.7955, p=0.0318). However, we did not observe any significant impact of immune cell densities or gp96 expression on patient outcomes. Finally, we revealed the association of MxA expression with prognosis linked to a preexisting TCR clone (CDR3-2) but was independent of the peripheral tumor-specific immune response. Taken together, low MxA expression correlated with better survival in GBM patients receiving HSPPC-96 vaccination, indicating MxA as a potential biomarker for early recognition of responsive patients to this immunotherapy.

**Clinical Trial Registration:** ClinicalTrials.gov (NCT02122822) http://www. chictr.org.cn/enindex.aspx (ChiCTR-ONC-13003309).

## Introduction

Glioblastoma multiforme (GBM) is one of the most lethal brain cancers and accounts for 48.6% of all primary brain malignancies (1), posing a great threat to human health, as current therapies are minimally effective (2). Initial surgical resection, adjuvant radiotherapy and chemotherapy with temozolomide constitute the standard-of-care therapy for GBM; however, they yield only a moderate increase in survival, with a reported median overall survival (OS) of 14.6 months (3) and a 5-year survival rate of less than 10% (4). Therefore, new therapeutic modalities are urgently needed to improve the outcomes of patients with this deadly brain cancer.

Recent advances in immunotherapies, such as immune checkpoint blockade, have brought substantial improvement in survival for a variety of solid malignancies, including melanoma (5), non-small-cell lung cancer (6), breast cancer (7), and non-Hodgkin lymphoma (8). In treating GBMs, several novel immune approaches are under development and have generated encouraging results in preclinical studies (9, 10) as well as in early trials (11, 12). Heat shock protein glycoprotein 96 kDa (gp96) belongs to the heat shock protein family, mainly locates in the endoplasmic reticulum (ER) and where it functions as a master chaperone. Gp96 has innate capacity of binding tumor-associated antigens (peptides), thereby forming a gp96-peptides complex that can be taken up by antigen-presenting cells, such as dendritic cells, and then elicit both innate and adaptive antitumor immune response (13). Therefore, after simple purification of the complex from patient tumors, the gp96-peptides complex can be exploited as a personalized multivalent cancer-treatment vaccine, usually termed as heat shock protein peptide complex 96 (HSPPC-96) (14). HSPPC-96 has exhibited its safety and preliminary clinical efficacy in treating a variety of malignancies (15–19), that include recurrent (20) and newly-diagnosed GBMs (21, 22). Our previous phase 1 clinical trial has demonstrated the safety and preliminary effectiveness of the heat shock protein peptide complex 96 (HSPPC-96) vaccine in treating newly diagnosed GBM patients (21). However, similar to other immunotherapies in solid tumors (16, 23), the efficacy of HSPPC-96 vaccination varies greatly, with OS times ranging from 7.5 months to 68.2 months in this cohort of patients (24). Therefore, the discovery of biomarkers that facilitate the recognition of patients who are likely to respond to immunotherapy is paramount. We found that the post-vaccination tumor-specific immune response (TSIR-post_vac), measured by an IFN-γ-releasing enzyme-linked immunospot (ELISPOT) assay on peripheral blood mononuclear cells (PBMCs), was associated with patient survival time (21). Higher TSIR-post_vac levels predicted better outcomes (21). We utilized second-generation T cell receptor (TCR) sequencing to examine TCR repertoire features in tumors and revealed the presence of some TCR clones predicting durable survival from HSPPC-96 vaccination (24). However, neither the ELISPOT assay nor TCR sequencing is a common clinical method, which limits the wide application of the aforementioned biomarkers.

Since protein detection methods, such as immunohistochemical (IHC) staining and enzyme-linked immunosorbent assay (ELISA), are routinely used in the clinical setting, protein marker candidates have more opportunities for translation from bench to bedside. In this study, we applied a multidimensional immunofluorescence (MIF) method to detect the infiltrative levels of immune cells (CD4^+^, CD8^+^ and PD-1^+^ immune cells) and the protein expression of MxA (encoded by an interferon-stimulating gene MX1) and gp96 (glycoprotein 96, a major component of HSPPC-96 vaccine) in pre-vaccination GBM tissues and examined their value in predicting the therapeutic outcomes of HSPPC-96 vaccination. We hypothesized that the levels of these biomarker candidates would reflect a natural state of antitumor response within tumors that would correlate with immunotherapeutic outcomes because they have been reported to directly engage adaptive/innate antitumor immune responses (25–28), and some have been used as outcome-predictive biomarkers for other cancer immunotherapies (29–31). We observed that low MxA expression correlated with better outcomes in this HSPPC-96 vaccinated cohort, reflecting the potential of MxA as a protein biomarker for the early recognition (prior to vaccination) of responsive patients to this immunotherapy.

## Materials and Methods

### Patients

We retrospectively examined the expression of the studied proteins by using formalin-fixed and paraffin-embedded (FFPE) tumor tissues that were collected during neurosurgical resection (prior to vaccination) from a cohort of 19 GBM patients receiving HSPPC-96 vaccination in an open-label, single-arm, phase I clinical trial (21). The trial was aimed at determining the safety and preliminary effectiveness of HSPPC-96 vaccination in treating newly diagnosed GBMs with standard-of-care therapy (21). After postoperative concurrent chemoradiotherapy, all the included patients received a total of six doses of the vaccine, with a 25-μg dose every week. The vaccine was generated through extracting the gp96 and its binding peptides from fresh tumor tissue according to the procedure described previously (21, 32). Cure & Sure Biotech Co. Ltd. was responsible for the vaccine production, following good manufacturing practice guidelines. A total of 20 patients were vaccinated in this trial; 19 patients with complete follow-up information were included for survival analysis, yielding a median progression-free survival (PFS) of 11.0 months and a median OS of 31.4 months in these patients (21). We continued follow-up with all survivors approximately 1 year after the end of the trial and updated the survival data, yielding a 20% PFS and a 40% OS at 3 years for all vaccinated patients (24). The clinical trial was approved by the ethics committee of Beijing Tiantan Hospital (JS2012-001-03) and was registered at ClinicalTrials.gov (NCT02122822) and http://www.chictr.org.cn/enindex.aspx (ChiCTR-ONC-13003309). Written informed consent was obtained from all participants.

The baseline characteristics of the included patients, such as sex, age at diagnosis, Karnofsky performance status (KPS) scale (range 0-100%), MGMT promoter methylation status, IDH 1/2 mutations and TERT promoter mutations, were reported by our previous study (21) and are shown in **Supplemental Table 1**.

### MIF and Image Analysis

We used OPALTM 7-color Manual IHC kits (NEL811001KT, Akoya Bioscience, Marlborough, MA, USA) to conduct MIF on the FFPE tissues. After deparaffinization and rehydration, the slides were subjected to a procedure that was optimized for each antigen. The experimental details are summarized in **Supplemental Table 2**.

Images were obtained on a Vectra system (Vectra Polaris 1.0.7, Akoya Bioscience, Marlborough, MA, USA) and analyzed by Inform software (2.4.2, Akoya Bioscience, Marlborough, MA, USA). Densities of infiltrating immune cells (CD4^+^, CD8^+^ and PD-1^+^ cells) were semi-quantified as counts per mm^2^ of tumor area. The expression of gp96 and MxA was semi-quantified as the staining extent, which was defined as the number of nuclei of positively stained cells divided by the number of all nuclei in the section. The counts and staining extent were automatically calculated by Inform software and then manually adjusted by an experienced pathologist.

### Statistical Analysis

All statistical analyses were performed using SPSS software version 26.0 (IBM, New York, United States). Continuous data were compared using the Mann–Whitney U test or Kruskal–Wallis nonparametric test. Spearman correlation analysis was used to examine the associations. Kaplan–Meier analysis was used to estimate PFS and OS, and the log-rank test was applied to estimate between-group PFS/OS differences. A Cox regression model was fitted to select independent prognostic factors. A 2-tailed P value of less than 0.05 was considered significant. GraphPad Prism 6 (GraphPad Software, Inc., San Diego, CA) was used to plot the figures.

## Results

### Immune Cell Densities and MxA/gp96 Expression Varied Greatly Among GBM Tissue Samples

Our previous study demonstrated that HSPPC-96 vaccination is a safe and preliminarily effective immunotherapy for the treatment of newly diagnosed GBMs (21). Despite these encouraging results, clinical outcomes remain highly variable, such that a fraction of patients did not benefit from the therapy compared with the standard treatment (21, 24). Therefore, the identification of patients who are more likely to obtain a survival benefit prior to vaccination is vital for this immunotherapy. We hypothesized that the immune features within the tumor microenvironment would contain predictive biomarkers for treatment efficacy, since the clinical efficacy of vaccines partly relies on boosting a pre-existing intra-tumor antitumor immune response (33, 34), which would be influenced by these immune features.

**Figure 1:**
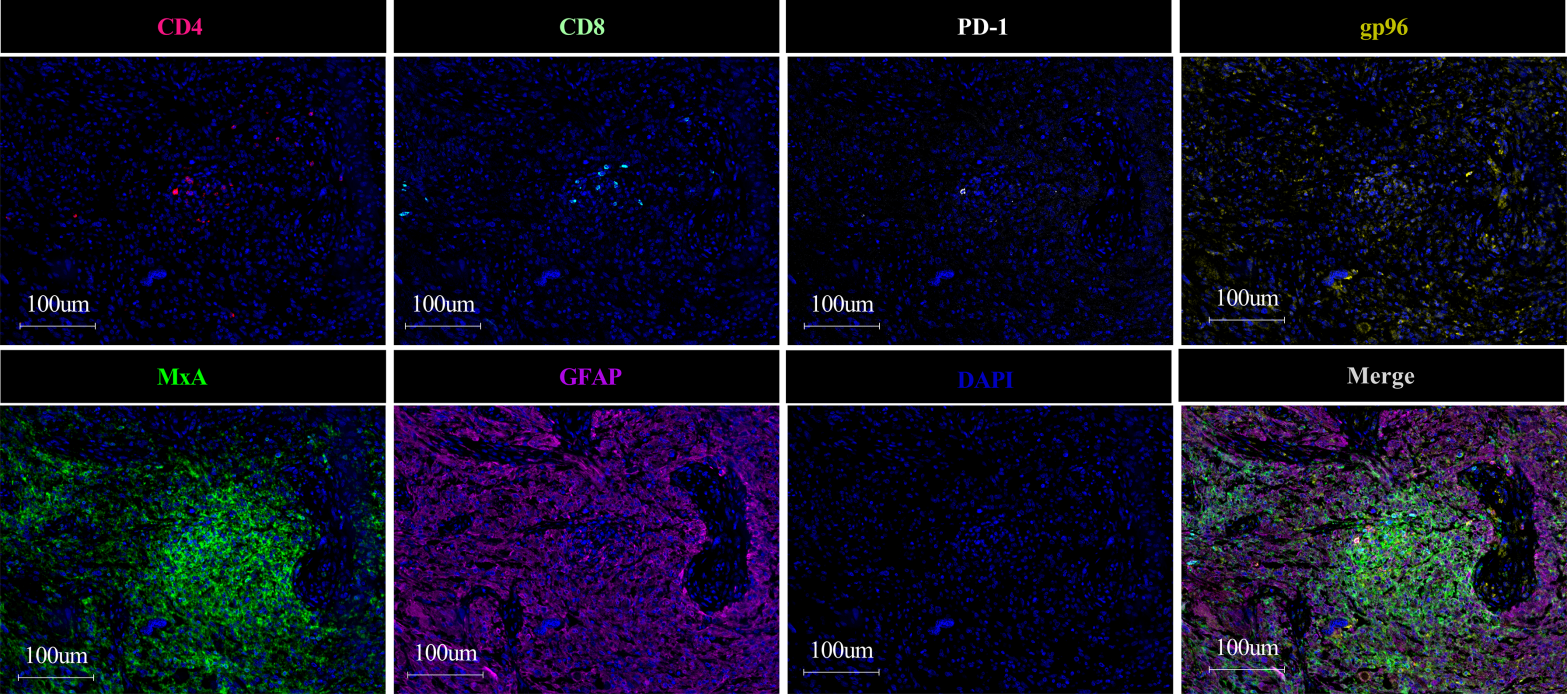
Representative staining of CD4+, CD8+, and PD-1+ cells and gp96 and MxA in GBM tissues.

Herein, we utilized MIF to detect immune features, including densities of immune cells (CD4^+^ T cells, CD8^+^ T cells and PD1^+^ cells) and protein expression of gp96 (a component of the HSPPC-96 vaccine) and MxA (an interferon-stimulating gene product, whose expression reflects interferon-pathway activity) (**Figure 1**), in FFPE tumor samples that were collected prior to vaccination from all 19 included patients with complete follow-up (24). Consistent with the findings of previous studies (35), the infiltrative levels of immune cells among samples varied greatly, with median levels of 78/mm^2^ (range 2-356/mm^2^), 20/mm^2^ (range 1-185/mm^2^) and 14/mm^2^ (range 1-101 mm^2^) for CD4^+^ T cells, CD8^+^ T cells and PD1^+^ cells, respectively. We also observed higher infiltration of CD4^+^ T cells than CD8^+^ T cells (**Supplemental Figure 1**) (36, 37). Both gp96 and MxA were detected by cytoplasmic staining (**Figure 1**) and were ubiquitously expressed in all the samples. However, the extent of staining in each sample also varied significantly among samples, with ranges of 2.2%-22% and 1.5%-49% for gp96 and MxA, respectively. These results indicate a significant variance in immune features among the tumor samples prior to vaccination, which provides a basis for further analyzing their impacts on clinical outcomes.

### Low MxA Expression Was Associated With Favorable Prognosis

**Figure 2:**
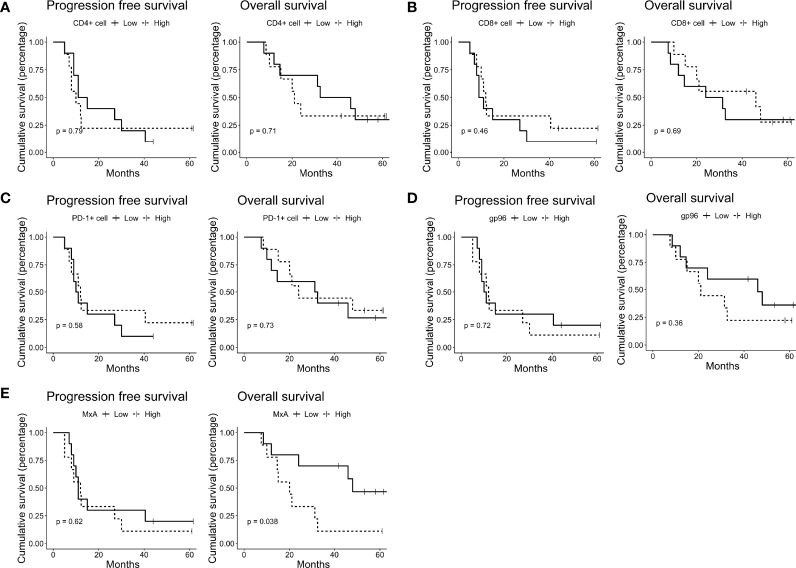
Low MxA expression was associated with favorable prognosis. Kaplan–Meier estimates of progression-free survival and overall survival in HSPPC-96- vaccinated GBM patients, grouped by the median CD4+ **(A)** CD8+ **(B)** and PD-1+ cell **(C)** densities and gp96 **(D)** and MxA **(E)** expression. Log-rank tests were applied to estimate differences. Vertical lines indicate censored time points.

We next explored the potential of these immune features as predictive biomarkers of treatment efficacy. We used the updated follow-up data that were published previously (24). After a median follow-up of 58.9 months, the median PFS and OS were 11.0 months and 31.4 months, respectively; PFS was 20% (4/20) and OS was 40% (8/20) at 3 years for all 19 vaccinated patients (24). According to the median levels of these immune features, we separately grouped the patients into the high and low groups. We did not observe a significant impact of the densities of immune cells, including CD4^+^ T cells, CD8^+^ T cells and PD1^+^ cells, on the survival of these patients (**Figures 2A–C**). However, high gp96 expression tended to be negatively correlated with patient OS (**Figure 2D**), although the correlation did not reach a significant level (p=0.364) due to the limited sample size. Interestingly, we observed that the expression of MxA negatively impacted the OS of these patients, with the MxA-low expression group exhibiting a median OS of 48 months compared with 20 months in the MxA-high expression group (p=0.038) (**Figure 2E**). This finding indicates the potential of MxA as a predictive biomarker for HSPPC-96 vaccination efficacy.

### Low MxA Expression Was Associated With Long-Term Survival

**Figure 3:**
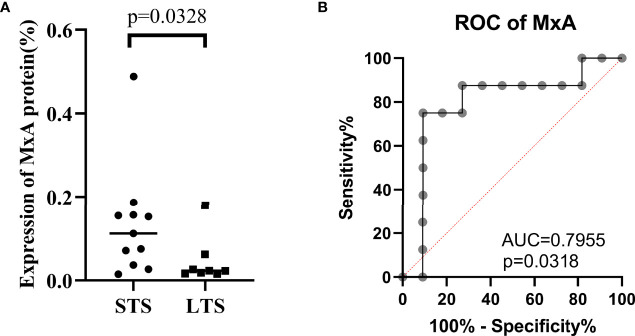
Low MxA expression was associated long-term survival. Comparison of MxA protein expression between long-term survivors (LTS, ≥3-y overall survival) and short-term survivors (STS, <3-y overall survival), Wilcoxon nonparametric test **(A)**. Receiver operating characteristic (ROC) curve analysis for evaluating the capacity of MxA protein expression to distinguish between LTS and STS **(B)**.

We grouped the vaccinated patients into long-term survivors (LTS) and short-term survivors (STS) according to whether their survival time was over three years, a common cut-off in survival to define LTS in GBM clinical studies (38, 39). Among the 19 included patients, 8 patients were LTS, and 11 were STS. As expected, the LTS group exhibited significantly lower MxA expression than the STS group (P = 0.0328) (**Figure 3A**). Furthermore, receiver operating characteristic (ROC) curve analysis indicated that MxA expression is a good indicator for distinguishing LTS and STS (AUC=0.7955, P=0.0318) (**Figure 3B**). Since LTS is a subset of patients who are most likely responsive to immunotherapy, these results indicate that MxA could be a potential biomarker for the early recognition of these responders prior to vaccination initiation.

### Association of MxA Expression With Prognosis Was Linked to a Preexisting TCR Clone But Was Independent of TSIR

**Figure 4:**
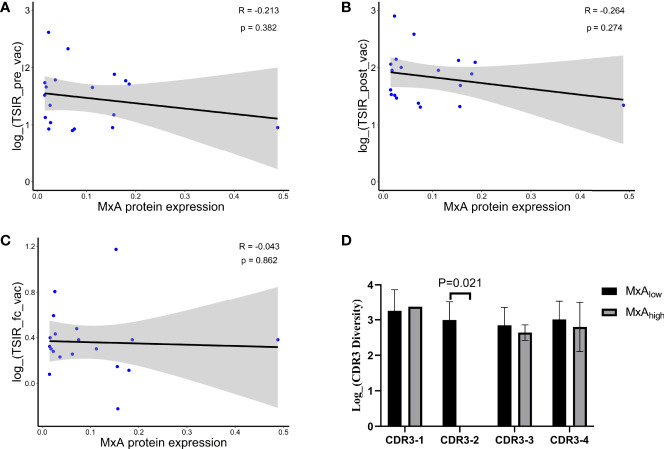
Association of MxA expression with prognosis linked to a preexisting TCR clone but was independent of TSIR. Pearson correlation of the expression of MxA protein with TSIR pre-vaccination **(A)**, TSIR post-vaccination **(B)**, and fold change of TSIR from pre- to post-vaccination **(C)** in 19 vaccinated patients. **(D)** Comparison of TCR clonotypes specific for long-term survivors between the high MxA protein expression group (MxA high: expression of MxA protein > the median value) and the low MxA protein expression group (MxA low: expression of MxA protein ≤ the median value), Wilcoxon nonparametric test. Continuous data were compared using the Wilcoxon nonparametric test.

We next investigated the underlying mechanisms that link low expression to better clinical outcomes. We previously used an ELISPOT assay to evaluate the TSIR levels in PBMCs collected before and after HSPPC-96 vaccination (21). We observed that HSPPC-96 vaccination increased TSIR levels by 2.3-fold, and the level of TSIR post-vaccination was closely associated with patient survival (21). Based on these observations, we first reasoned that low MxA expression could be associated with increased levels of TSIR post-vaccination, thereby leading to favorable outcomes. However, we did not observe any significant correlation between MxA expression and the levels of three TSIR indexes, including TSIR pre-vaccination (TSIR_pre_vac, **Figure 4A**), TSIR post-vaccination (TSIR_post_vac, **Figure 4B**) and fold change of TSIR from pre- to post-vaccination (TSIR_fc_vac, **Figure 4C**). Moreover, a fitted Cox regression model, including age, TSIR_post_vac and MxA expression, also revealed MxA expression as an independent prognosticator of outcomes (**Supplemental Figure 2**). Therefore, MxA expression and TSIR_post_vac independently influenced the outcomes in this vaccinated cohort.

We used TCR sequencing to uncover the TCR repertoire features of tumor-infiltrating lymphocytes in LTS and discovered that four TCR clones were significantly enriched in the LTS group, and the presence of these clones was associated with favorable outcomes (24). Therefore, we next explored whether the link of MxA expression to outcomes was associated with the unbalanced distribution of these TCR clones. We compared the frequencies of these clones, represented as complementarity determining region 3 (CDR3), which determines the specificity of a given TCR clone, between the high- and low-MxA expression groups. As a result, we found an absence of CDR3-2 in the high-MxA expression group (p=0.021) (**Figure 4D**). Since the presence of CDR3-2 was associated with long-term survival in this cohort as well as in another peptide vaccine trial on gliomas (33), we reasoned that the negative impact of MxA expression on CDR3-2 presence could be related to its inverse correlation with patient survival time. However, given the limited sample size of this study, this impact should be further validated in another HSPPC-96 vaccinated cohort with a larger population. The intrinsic mechanism remains unclear and requires further exploration.

## Discussion

Immunotherapy has emerged as a primary therapeutic option for a variety of malignancies (40–44) and has exhibited encouraging results in early trials on GBMs (45–49). HSPPC-96 is a personalized multivalent antitumor vaccine that has demonstrated its low toxicity and promising clinical results for the treatment of a variety of malignancies in preclinical models (50, 51) as well as early-phase trials (15, 18, 52). However, the high variance in immunotherapeutic efficacy remains one of the biggest challenges facing this immunotherapy (20, 53). In our HSPPC-96 vaccinated cohort, the OS time varied greatly, ranging from 7.5 to 68.2 months (24). Thus, the identification of patients who are more likely to respond to or benefit from immunotherapy is vital for this novel therapeutic modality (24). Although the lack of correlation of clinical activity to immune responses has been widely noted (33, 54–58), we still used the ELISPOT assay (21, 24) and TCR sequencing (24) to discover the biomarkers related to immune responses for predicting therapeutic outcomes of HSPPC-96 vaccination. We observed that TSIR post-vaccination (21, 24) and a group of TCR clones (24) were potential biomarkers for predicting better clinical outcomes. Nevertheless, the timing of detecting TSIR post-vaccination (prohibiting its early recognition), the cost of TCR sequencing and the unavailability of both in a routine clinical setting all limit the wide application of these biomarkers. In this study, we aimed to discover protein biomarkers in tumor tissues for the recognition of patients who would respond to or benefit from HSPPC-96 vaccination at an early time prior to vaccination. We found that MxA is a potential biomarker for the prior-to-vaccination recognition of responsive patients. Since MxA can be detected *via* the method of immunohistochemical staining on FFPE tissues, which has been widely and routinely used for clinical diagnosis, the discovered marker would be more easily translated from bench to bedside.

MxA belongs to a family of large GTPases and has been extensively studied for its broad antiviral activity (59, 60). It is exclusively induced by interferon-α/β and represents a classical interferon-stimulating gene product (61–63). Therefore, a large number of studies applied MxA expression as an indicator for measuring the activity of the interferon-α/β signalling pathway (62, 63). Meanwhile, recent findings have suggested MxA as an oncoprotein in breast cancer (26), as it promotes tumor cell invasion and proliferation. In this study, we found that high MxA expression deteriorated the immunotherapeutic efficacy of HSPPC-96 vaccination (**Figure 2E**). With respect to the underlying mechanisms explaining the detrimental effect of MxA expression, we speculate that high MxA expression reflects a strong autocrine activation of the interferon-α/β signaling pathway (64) that has been proven to dampen the antitumor immune response by impairing the immunogenicity of glioma cells (61). Additionally, the pro-tumor effect of MxA (26, 65) itself would also mediate its negative impact on the vaccination effectiveness. Therefore, this result also suggests a possible mechanism for the immune evasion of GBM cells against HSPPC-96 vaccination, thus providing possible molecular targets to be manipulated for further improving the therapeutic efficacy.

Nevertheless, we did not find any evidence that high MxA expression suppressed the antitumor immune response in peripheral blood (**Figures 4A–C**), suggesting that the immune evasion mechanism is not exerted at the early stage when antitumor T cells are activated in blood, but could be at the late stage after these T cells infiltrate the tumors. Interestingly, we found that high MxA expression was associated with loss of a TCR clone, CDR3-2 (**Figure 4D**), that predicted a durable survival in glioma patients receiving therapeutic peptide vaccination (24, 33). Since this TCR clone, shared by LTS, could reflect a pre-existing T cell-mediated immune response against GBM cells (24), we speculated that the loss or downregulation of the pre-existing antitumor immune response within tumors could contribute to the inverse correlation of high MxA expression with poorer outcomes. However, the underlying mechanisms remain elusive, requiring further exploration.

In this study, we also investigated whether the pre-existing levels of T cell infiltration could affect the immunotherapeutic efficacy of HSPPC-96 vaccination, since they are widely recognized as indicators predicting patients’ response to immunotherapies, such as immune checkpoint blockade (11, 66, 67). However, we did not observe any correlation of T cell infiltration levels, in terms of CD8^+^, CD4^+^ and PD-1^+^ cell densities, with clinical outcomes (**Figures 2A–C**). Considering that the presence of some TCR clones was linked to better immunotherapeutic outcomes in this cohort (24), these results suggest that the amount of a subset of, rather than all, the infiltrative T cells, impacts the vaccination outcomes.

As a major component of the HSPPC-96 vaccine, gp96 is a molecular chaperone in the endoplasmic reticulum (ER) that has been reportedly linked to maintaining the ER stability (68), mediating unfolded protein responses (69, 70), promoting tumor invasiveness (71) as well as facilitating activation of adaptive and innate immune responses (72). We observed an interesting trend of high gp96 expression linked to unfavorable OS in this cohort (p=0.364), although it did not reach a significant level given the limited sample size. This result indicates an inverse correlation between the natural expression of gp96 and effectiveness of the HSPPC-96 vaccine, a vaccine comprising gp96 and its binding antigenic peptides. This correlation suggests a possible interplay between gp96 expression and an antitumor immune response that is required for the therapeutic effectiveness. Therefore, this correlation and its intrinsic mechanism require further investigation in clinical studies on a larger cohort of patients as well as intensive preclinical studies.

Again, although the correlations of MxA expression with the immunotherapeutic outcomes as well as the CDR3-2 TCR clone are statistically significant, these findings warrant further confirmation in another larger prospective cohort, given the small sample size and retrospective design of this study.

## Conclusions

In conclusion, we revealed that low expression of MxA correlated with better survival in GBM patients receiving HSPPC-96 vaccination, indicating that MxA is a potential biomarker for the pre-vaccination recognition of responsive patients to this immunotherapy.

## Data Availability Statement

The raw data supporting the conclusions of this article will be made available by the authors, without undue reservation.

## Ethics Statement

The studies involving human participants were reviewed and approved by the ethics committee of Beijing Tiantan Hospital. The patients/participants provided their written informed consent to participate in this study.

## Author Contributions

Conceptualization, YZ and NJ; Data curation, YW and CL; Formal analysis, YW and YZ; Funding acquisition, YZ and NJ; Methodology, YW and CL; Project administration, YZ and NJ; Resources, NJ; Software, YW and XC; Supervision, YZ and NJ; Validation, XH and HG; Visualization, YW; Writing – original draft, YW; Writing – review and editing, YZ and NJ. All authors have read and agreed to the published version of the manuscript.

## Funding

National Natural Science Foundation of China (81702451, 81930048), Capital characteristic Clinical Application Project (Z181100001718196), the capital health research and development of special (2022-2-2047).

## Conflict of Interest

Authors XH and HG are employed by Cure & Sure Biotech Co., LTD, Shenzhen, China, 518000.

The remaining authors declare that the research was conducted in the absence of any commercial or financial relationships that could be construed as a potential conflict of interest.

## Publisher’s Note

All claims expressed in this article are solely those of the authors and do not necessarily represent those of their affiliated organizations, or those of the publisher, the editors and the reviewers. Any product that may be evaluated in this article, or claim that may be made by its manufacturer, is not guaranteed or endorsed by the publisher.
