# Supplementary material for: Low MxA Expression Predicts Better Immunotherapeutic Outcomes in Glioblastoma Patients Receiving Heat Shock Protein Peptide Complex 96 Vaccination
Source: Front Oncol. 2022 Jul 12;12:865779. doi: 10.3389/fonc.2022.865779 (PMC9321638; doi:10.3389/fonc.2022.865779)

Supplementary Material

**Supplemental Table 1. Basic characteristics of all treated patients.**

| **Variables** | **Number** | **Proportion (%)** |
| --- | --- | --- |
| **Gender** |  |  |
| Male | 9 | 47.3 |
| Female | 10 | 52.7 |
| **Median age at diagnosis, yr (range)** 52 (40-70) | | |
| **Median preoperative KPS (range)**  80 (70-100) | | |
| **MGMT promoter status** |  |  |
| Methylation | 2 | 10.5 |
| Unmethylation | 14 | 73.7 |
| Unknown | 3 | 15.8 |
| **IDH 1/2 mutations** |  |  |
| Mutant | 2 | 10.5 |
| Wild-type | 14 | 73.7 |
| Unknown | 3 | 15.8 |
| **TERT promoter mutations** |  |  |
| Mutant | 11 | 57.9 |
| Wildtype | 5 | 26.3 |
| Unknown | 3 | 15.8 |
| **Status at last follow-up** |  |  |
| Alive | 6 | 31.5 |
| Dead | 13 | 68.5 |
| **Median follow-up time, mo (range)** 58.9 (34.6-80.2) | | |

KPS, Karnofsky performance score; MGMT, O6-methylguanine-DNA methyltransferase; IDH, isocitrate dehydrogenase.TERT, Telomerase reverse transcriptase.

**Supplemental Table 2. Detailed experimental conditions of the MIF assay.**

| **Supplement Table 2A: Primary antibodies used in the multiplex immunofluorescence assay.** | | | | | |
| --- | --- | --- | --- | --- | --- |
| **Stain Order** | **Antibody** | **Clone or lot# (host)/Company** | **Dilution** | **Opal dye (Dilution)** | **CITY/Nation** |
| **1** | CD8 | ab4055(Rabbit)/Abcam | 1/400 | 540 (1:100) | Cambridge/UK |
| **2** | PD-1 | NAT105 (Mouse)/Abcam | 1/400 | 650 (1:100) | Cambridge/UK |
| **3** | Gp96 | ab3674 (Rabbit)/Abcam | 1/400 | 570 (1:100) | Cambridge/UK |
| **4** | GFAP | EPR1034Y (Rabbit)/Abcam | 1/400 | 690 (1:100) | Cambridge/UK |
| **5** | CD4 | EPR6855 (Rabbit)/Abcam | 1/400 | 620 (1:100) | Cambridge/UK |
| **6** | MxA | ab95926 (Rabbit)/Abcam | 1/200 | 520 (1:100) | Cambridge/UK |
| **7** | DAPI | C0060/Solarbio | 1/500 |  | Beijing/China |

| **Supplement Table 2B: Experimental conditions and procedures for the multiplex immunofluorescence assay.** | | | | | | | |
| --- | --- | --- | --- | --- | --- | --- | --- |
| **Antigen** | **CD8** | **PD-1** | **Gp96** | **GFAP** | **CD4** | **MxA** | **DAPI** |
| **Antigen retrieval** | AR6  20 min | AR6  20 min | AR9  20 min | AR6  20 min | AR9  20 min | AR6  20 min |  |
| **Wash** | TBST  10 min | TBST  10 min | TBST  10 min | TBST  10 min | TBST  10 min | TBST  10 min |  |
| **Blocking** | 10 min  @RT | 10 min  @RT | 10 min  @RT | 10 min  @RT | 10 min  @RT | 10 min  @RT |  |
| **Inc. with Primary antibody** | 60 min  @37℃ | Over night  @4℃ | Over night  @4℃ | 60 min  @37℃ | Over night  @4℃ | Over night  @4℃ | 10 min  @RT |
| **Wash** | TBST  10 min | TBST  10 min | TBST  10 min | TBST  10 min | TBST  10 min | TBST  10 min | TBST  10 min |
| **Inc. with Opal HRP Polymer Ms+Rb** | 10 min  @RT | 10 min  @RT | 10 min  @RT | 10 min  @RT | 10 min  @RT | 10 min  @RT |  |
| **Wash** | TBST  10 min | TBST  10 min | TBST  10 min | TBST  10 min | TBST  10 min | TBST  10 min | Mounting |
| **Inc. with Opal Dye** | opal 540  10 min  @RT | opal 650  10 min  @RT | opal 570  10 min  @RT | opal 690  10 min  @RT | opal 620  10 min  @RT | opal 520  10 min  @RT |  |
| **Wash** | TBST  10 min | TBST  10 min | TBST  10 min | TBST  10 min | TBST  10 min | TBST  10 min |  |
| Abbreviations: AR6: Antigen retrieval at pH=6; AR9: Antigen retrieval at pH=9; @; at; RT: at Room temperature; Inc: Incubation; Temp: Temperature; Ms+Rb: Mouse and Rabbit | | | | | | | |

**Supplemental Figure 1. Higher infiltration of CD4^+^ T cells than CD8^+^ T cells.**


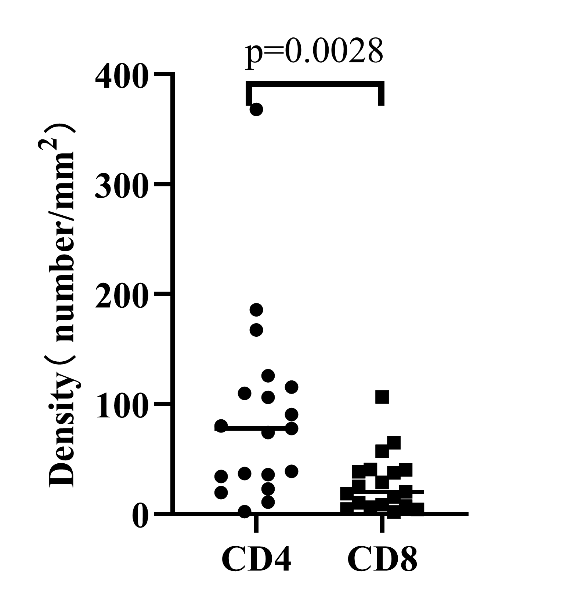


**Supplemental Figure 2. A Cox regression model predicting median overall survival**


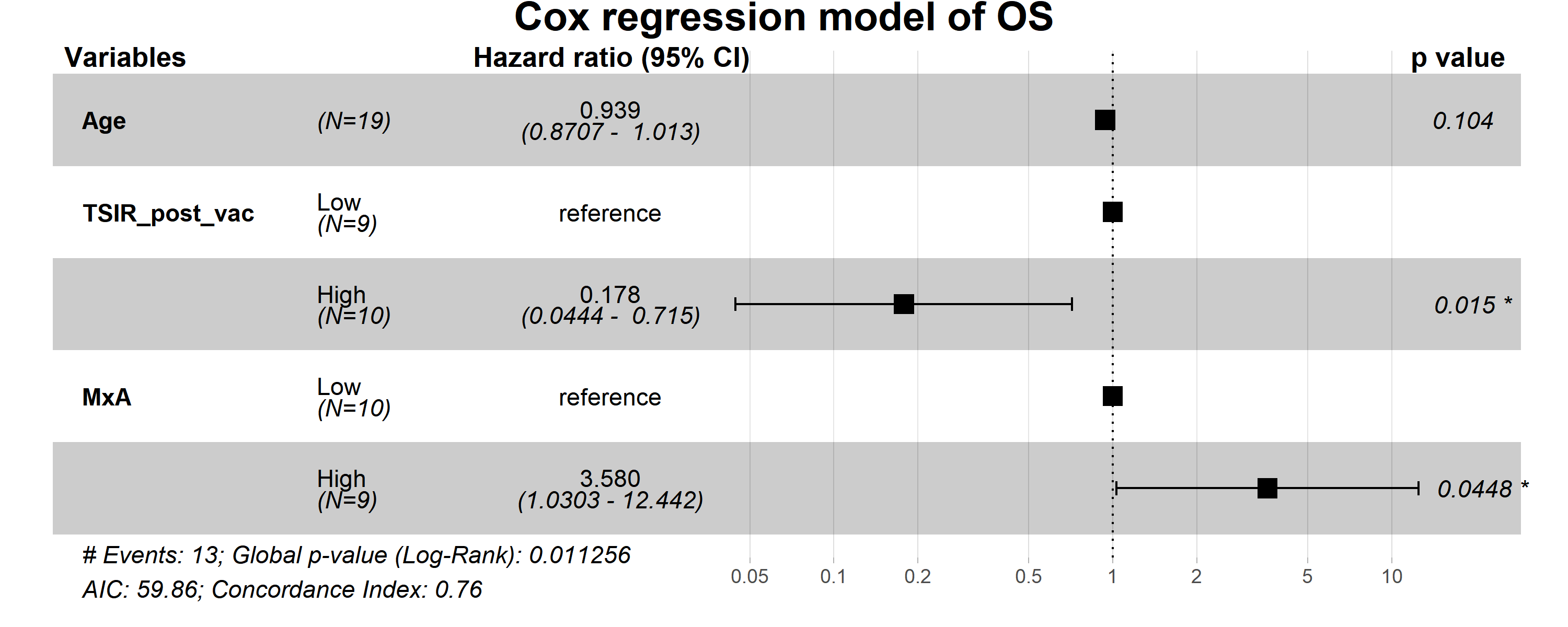

Supplement: Supplementary file 1 [file DataSheet_1.docx]
